# The problem with an Hepatic Artery Injury Postlaparoscopic Cholecystectomy in China

**DOI:** 10.12669/pjms.301.4639

**Published:** 2014

**Authors:** Li Li, Bin Song, Zhoupeng Wu

**Affiliations:** 1 Li Li, MD, Department of Radiology, West China Hospital, Sichuan University, Guo xue xiang 37, Chengdu, Sichuan 610041, China.; 2 Bin Song, MD, Department of Radiology, West China Hospital, Sichuan University, Guo xue xiang 37, Chengdu, Sichuan 610041, China.; 3 Zhoupeng Wu, MD, Department of Vascular Surgery,West China Hospital, Sichuan University, China.

The incidence of vascular injury remains a problematic complication of laparoscopic cholecystectomy, due to the anatomic variations of the cystic artery. Therefore, the focus of this study was to investigate the origin, course, and termination of the cystic artery using dual source computed tomography.

Currently, laparoscopic cholecystectomy (LC) has emerged as a golden standard in the treatment of cholelithiasis. A hepatic artery remains a problematic complication of laparoscopic cholecystectomy. The diversity of the anatomical variations of the cystic artery increases the risk of injury during LC, particularly in the presence of acute inflammation.

It has been reported that a hepatic artery during laparoscopic cholecystectomy mainly occur during inadvertent procedure. Its incidence after cholecystectomy has been estimated to be 8% in an autopsy series of cadavers who had undergone laparoscopic cholecystectomy in China. This incidence seems increased in patients with a bile duct injury, ranging between 14% and 29%. As angiographic studies are usually not routinely performed, the exact figure is however unknown. It has been proposed that the presence of an hepatic artery may result in several complications including liver necrosis or abscess, increase the risk of bleeding at the time of the biliary repair, and favor recurrent stenosis.

The development of multi-detector row CT has made it possible to clearly depict the cystic artery. Takahashi S et al.^1^ found that four–detector row CT made the hepatic artery more visible, including cystic artery. Sugita R et al.^2^ addressed the course of the cystic artery regarding the relationship to the Calot triangle with 64–detector row CT. In their study, they also reported the relationship between the cystic arteries and the Calot triangle and information on the anatomy of the cystic duct.

Stewart L et al.^3^ published a discussion of the mechanisms to the right hepatic artery injury according to Stewart-Way classification. It is likely that the reason why the RHA injury lied on that is because the right hepatic artery is often mistaken for cystic artery (Stewart-Way class IV) (64%). But why the right hepatic artery mistaken for cystic artery is still not clear. Future research in China should focus on the anatomical details of the cystic artery and adjacent structures using dual source computed tomography (CT)
